# Hope for Tropical Biodiversity After All

**DOI:** 10.1371/journal.pbio.1002358

**Published:** 2016-01-19

**Authors:** Robin Meadows

**Affiliations:** Freelance Science Writer, Fairfield, California, United States of America

Much as Mark Twain famously stated that reports of his death were greatly exaggerated, it turns out that land animals in tropical protected areas may be better off than we thought. Current assessments warn of widespread biodiversity loss in the tropics—which is home to half of species globally—and put declines as high as 56% over the last four decades. But now, in this issue of *PLOS Biology*, Lydia Beaudrot, Jorge Ahumada, and colleagues report that tropical forest preserves around the world may be helping after all: at the community level, ground-dwelling mammals and birds are holding their own.

**Fig 1:**
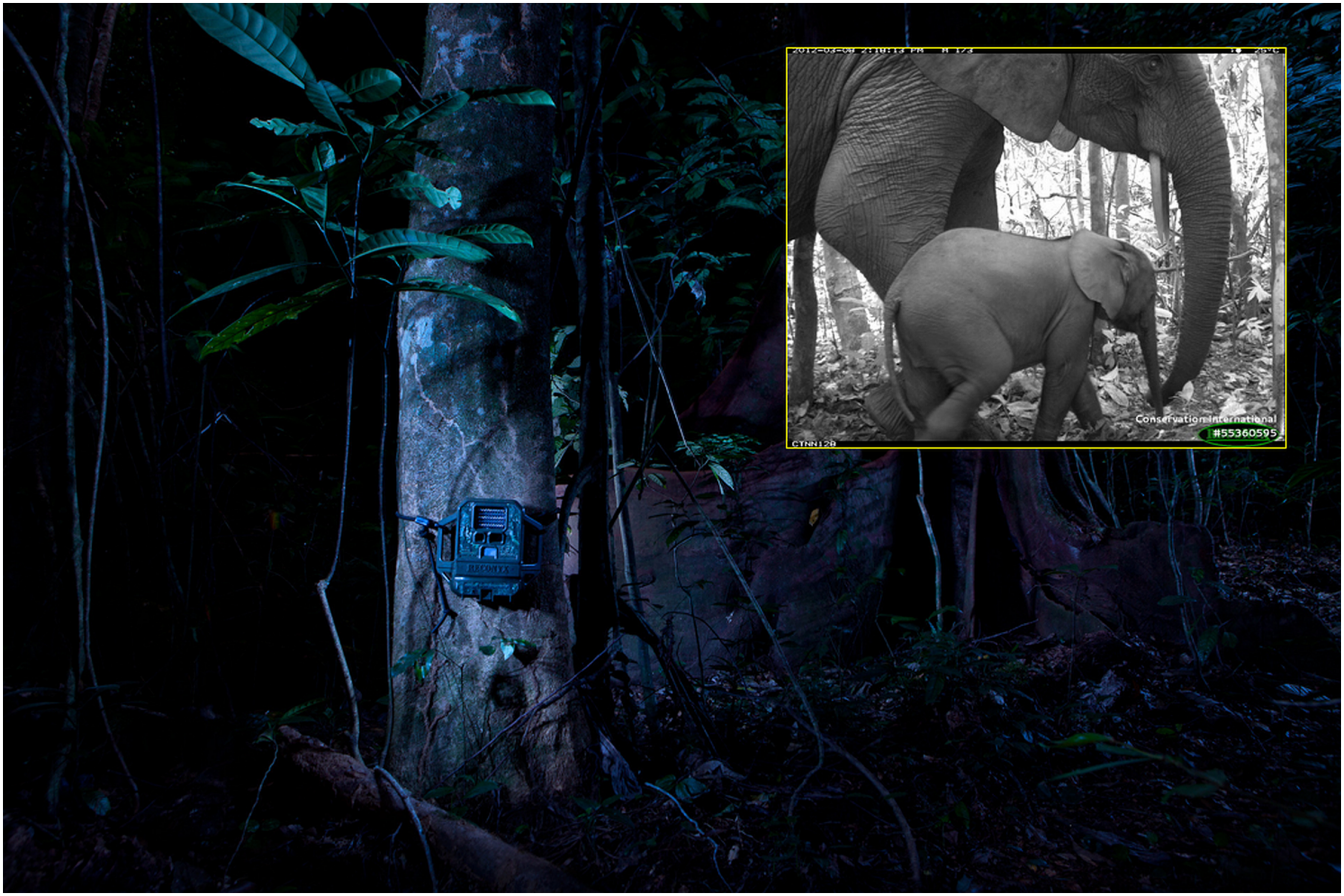
Camera traps in remote tropical forests around the world provide insight into the status of rare and endangered wildlife, such as these African bush elephants (*Loxodonta africana*). Photo credit: Benjamin Drummond and TEAM Network.

Why the difference? Tropical forests are understudied, and most of the animals living there are rare and elusive, so existing biodiversity assessments rely heavily on aggregated secondary data and expert opinion. In contrast, the new findings are based on standardized primary data from camera traps in the Tropical Ecology Assessment and Monitoring (TEAM) Network, which give close-to-real-time snapshots of wildlife status and trends (Fig 1).

Beaudrot, Ahumada, and colleagues determined biodiversity trends for more than 500 populations of nearly 250 species of mammals and birds, using half a million images per year from tropical forest preserves on three continents. The distribution of the 15 study sites reflected the proportion of tropical forest cover on each continent: half were in South and Central America, and the remainder were split between Africa and Southeast Asia. Each of the protected areas studied had a camera trap every square kilometer or two, for a total of 60 to 90 per site.

Even so, camera sightings were infrequent—five per year for a given population was not uncommon—and the researchers used occupancy as a proxy for abundance. Analysis of the sightings showed that occupancy declined in 22%, increased in 17%, and did not change in 22% of the populations during the study period, which ranged from three to eight years depending on the site.

Further analysis of the camera trap images revealed encouraging news: despite the variability in occupancy trends, biodiversity did not decline systematically at the community level in the protected areas studied. Importantly, occupancy trends did not vary significantly between mammals and birds, or by International Union for Conservation of Nature (IUCN) Red List status. The latter suggests that, at least in the study sites, populations of Threatened and Near Threatened species are doing as well as those of Least Concern.

However, sightings were too low to determine trends for nearly two-fifths of the populations studied, leading the researchers to caution that the observed community-level stability could mask wildlife losses in protected areas. The researchers also underscore that their findings do not apply to the many unprotected tropical forests worldwide. Today's extinction rates are estimated to be 1,000 times faster than the average over the history of life on Earth, and this catastrophic species loss occurs disproportionately in the tropics.

These caveats aside, this work gives hope that species loss in protected tropical forests is less severe than we feared. Moreover, for the first time, conservation decisions can be based on real-time changes in wildlife populations. Some 200 nations have committed to a suite of protection measures—increasing protected areas, preventing extinctions, and reversing wildlife declines—by 2020. This work provides governments and nonprofits with tools for identifying successful strategies to preserve the biodiversity that remains while we still can.
